# Impact of Bariatric Surgery on metabolic health in a Uruguayan cohort and the emerging predictive role of FSTL1

**DOI:** 10.1038/s41598-024-65651-8

**Published:** 2024-07-02

**Authors:** Leonardo Santos, Mariana Patrone, Victoria Prieto-Echagüe, Silvana Lapi, Mauro Perdomo, Andrea Vaucher, Gustavo Rodriguez, Pablo Valsangiacomo, Hugo Naya, Carlos Escande, Jose L. Badano, Lucia Spangenberg, Gustavo Bruno

**Affiliations:** 1 Laboratorio de Patologías del Metabolismo y El Envejecimiento, Institut Pasteur de Montevideo, Mataojo 2020, 11400 Montevideo, Uruguay; 2 Programa de Obesidad y Cirugía Bariátrica, Hospital Maciel, 25 Mayo 174, 11000 Montevideo, Uruguay; 3 Laboratorio de Genética Molecular Humana, Institut Pasteur de Montevideo, Mataojo 2020, 11400 Montevideo, Uruguay; 4 Clínica Quirúrgica 2, Facultad de Medicina, Universidad de la República (UDELAR), Gral Flores 2125, 11800 Montevideo, Uruguay; 5 Clínica Quirúrgica 3, Facultad de Medicina, Universidad de la República (UDELAR), Gral Flores 2125, 11800 Montevideo, Uruguay; 6 Clínica Médica 3, Facultad de Medicina, Universidad de la República (UDELAR), Gral Flores 2125, 11800 Montevideo, Uruguay; 7 Unidad de Bioinformática, Institut Pasteur de Montevideo, Mataojo 2020, 11400 Montevideo, Uruguay; 8 Departamento de Producción Animal y Pasturas, Facultad de Agronomía, Universidad de la República (UDELAR), Av. Gral. Eugenio Garzón 780, 12900 Montevideo, Uruguay; 9 Departamento Básico de Medicina, Hospital de Clínicas, Facultad de Medicina, Universidad de la República (UDELAR), Av Italia S/N, 11600 Montevideo, Uruguay

**Keywords:** Bariatric Surgery, FLST1, Linear models, Correlations, Obesity, Molecular biology, Data mining, Data processing

## Abstract

Obesity poses significant challenges, necessitating comprehensive strategies for effective intervention. Bariatric Surgery (BS) has emerged as a crucial therapeutic approach, demonstrating success in weight loss and comorbidity improvement. This study aimed to evaluate the outcomes of BS in a cohort of 48 Uruguayan patients and investigate the interplay between BS and clinical and metabolic features, with a specific focus on FSTL1, an emerging biomarker associated with obesity and inflammation. We quantitatively analyzed BS outcomes and constructed linear models to identify variables impacting BS success. The study revealed the effectiveness of BS in improving metabolic and clinical parameters. Importantly, variables correlating with BS success were identified, with higher pre-surgical FSTL1 levels associated with an increased effect of BS on BMI reduction. FSTL1 levels were measured from patient plasma using an ELISA kit pre-surgery and six months after. This research, despite limitations of a small sample size and limited follow-up time, contributes valuable insights into understanding and predicting the success of BS, highlighting the potential role of FSTL1 as a useful biomarker in obesity.

## Introduction

Obesity represents a pressing social and healthcare challenge, demanding comprehensive strategies to address its far-reaching implications. This pathology is a pervasive medical condition that profoundly affects human metabolism. Beyond its aesthetic concerns, obesity intricately disrupts metabolic homeostasis, giving rise to various health challenges^1–3^. In recent years, the escalating prevalence of obesity has encouraged extensive scientific research into the underlying metabolic disturbances associated with excess body weight^4–6^.

The treatment of obesity is comprehensive and multidisciplinary. Furthermore, the success of the treatment requires a high degree of motivation and planning. Therapeutic goals should be tailored to each patient based on the degree of obesity and associated risk factors. There are new therapeutic tools in the face of the failure of medical, behavioral, and nutritional treatments that achieve a healthy and sustainable metabolic state. Bariatric Surgery (BS) is one of the main therapeutic strategies for morbid obesity, and it is considered to be successful when it achieves 50–70% excess weight loss or 20–30% total weight loss or a BMI < 35 kg/m^2^^7,8^. BS reduces obesity-related comorbidities, improves quality of life, and decreases patient's risk of premature death by 30–50%^9,10^.

BS is recommended in patients with BMI > 35 kg/m^2^ regardless of the presence of comorbidities, in patients with BMI > 30 kg/m^2^ with type 2 diabetes, and also should be considered in individuals with a BMI of > 30 kg/m^2^ who do not achieve substantial or durable weight loss or co-morbidity improvement using nonsurgical methods. However, there is significant variability among patients, not only in terms of the presentation of obesity but also in how they respond to surgery, highlighting the importance of understanding the factors responsible for this variability.

The excessive accumulation of visceral adipose tissue during obesity is associated with changes in the profile of secreted molecules by this tissue, including adipokines (leptin, adiponectin), cytokines (IL-6, TNFalpha, IL1b), and other macromolecules and metabolites. The alteration in the secretion profile of adipose tissue largely contributes to the establishment of chronic inflammation and hormonal imbalances, which leads to insulin resistance and collectively increases the risk of chronic diseases such as diabetes, dyslipidemia, fatty liver, and cardiovascular diseases.

Although several circulating cytokines and adipokines have been correlated with establishing and progressing metabolic syndrome during obesity, some of these mechanisms remain poorly understood. Extensive research is currently focused on identifying physiologically relevant molecules and pathways that could serve as entry points to understand the cellular basis of obesity and as biomarkers to predict/monitor disease presentation and outcome. One such moiety is FSTL1 (Follistatin like 1). FSTL1 is a glycoprotein primarily produced by mesenchymal cells linked to various pathologies, including cardiovascular, autoimmune, neoplastic, osteoarticular conditions, and obesity (reviewed in^11^). FSTL1 is a secreted protein that circulates in the plasma, and therefore, it was suggested that it may function as adipokine/myokine (reviewed in^12^). Regarding functions directly related to obesity, FSTL1 was linked to adipogenesis by virtue of its presence in preadipocytes and significant downregulation in mature adipocytes^13^.

Our group showed that FSTL1 participates in the control of adipocyte differentiation in 3T3L1 cells and both its presence in preadipocytes as well as its reduction once adipogenesis is ongoing are important for adipocyte differentiation^14^. More recently, this function was confirmed in vivo in mice by others^15^. It has also been shown that FSTL1 plays an active role in the obesity-induced inflammatory response in both mice and humans^16,17^.

Importantly, different studies highlight the potential role of FSTL1 as a biomarker for various conditions, including obesity. For example, Horak and colleagues analyzed a cohort of 133 unrelated Czech individuals of caucasian origin, comparing FSTL1 plasma levels between 81 obese (BMI ~ 44.8) and 52 nonobese (BMI ~ 22.8) individuals. Interestingly, their study showed a significant downregulation of FSTL1 in morbidly and super obese individuals, and the authors suggest this decrease could be due to a reduction in adipogenesis, adipocyte apoptosis, and epigenetic silencing of the FSTL1 locus^18^. More recently, the work by Lee and colleagues sought to analyze the levels of circulating FSTL1 with the presentation of obesity, analyzing a cohort of 230 Korean individuals, comparing participants according to their BMI and metabolic health state. Interestingly, while they did not observe significant changes in FSTL1 levels based on BMI differences, they were able to associate high levels of FLST1 with a metabolic unhealthy state^19^. Of note, and regarding the association of FSTL1 levels with BMI, the results of these two studies are not necessarily contradictory. In addition to likely important genetic differences between cohorts, the mean BMI in the Korean patients considered as obese ranged between 26.7 and 27.2, compared to approximately 44.8 in the previous study. Importantly, the two studies did provide important data supporting the role of FSTL1 as a biomarker in obesity. However, further research is needed to fully understand the role of FSTL1 in this context and, therefore, its association with disease onset and presentation.

Here, we aim to assess the impact of BS on the clinical and metabolic features of a Uruguayan cohort of 48 patients who are part of the Program of Obesity and Bariatric Surgery (POBS) of the Maciel Hospital in Uruguay. In agreement with the literature, our results clearly show the multiple benefits of BS on obese patients. Additionally, we constructed linear models to identify clinical parameters (including FSTL1 levels) associated with BS success. Importantly, we identified different variables, including pre-surgical FSTL1 levels, that correlate with and were predictors of BS outcome. Our results, therefore, present novel tools to evaluate BS while further supporting the potential use of FSTL1 as a biomarker in obesity.

## Material and methods

### Ethical statement

This project was approved by the ethical board of Maciel University Hospital (Clinica Medica 3). All participants gave written informed consent. Research has been performed in accordance with the Declaration of Helsinki.

### Patient population and eligibility criteria

A prospective and observational study was carried out on a cohort of patients belonging to the Obesity and Bariatric Surgery Program (OBSP) of the Maciel Hospital. A random selection of patients from OBSP were recruited during the preoperative evaluation from July 2020 to July 2023. They were studied again six months after surgery, performing a clinical examination and drawing blood samples for analytical studies. The inclusion criteria for the OBSP are age between 18 and 65 years, BMI > 40 kg/m^2^ or BMI > 35 kg/m^2^ with comorbidities, and having failed medical-nutritional treatment. The exclusion criteria are addictions (smoking, alcoholism, or consumption of drugs of abuse), decompensated psychiatric pathology, lack of family support, or intellectual disability. Patients were recruited during the pre-operative assessment.

Table 1 includes some of the clinical data included in the study. Additional clinical data and post-surgical values that were assessed six months after surgery are included in Table S1. Uricemia, albumina, fasting glucose, Hb1Ac and hemoglobin levels were assessed by the laboratory of Clinical Analysis of Maciel Hospital. Fasting glucose levels required 8 h of fasting time.

**Table 1:** Clinical variables at preoperative evaluation.

	Sex	Age	wg	hg	BMI	BP sis	BP dia	HT	DM2	Uri	Alb	Glu	Hb1Ac	HB
Pre surgery														
p1	1	51	134	1.52	56.5	160	100	0	0	3.9	4.11	0.82	6.5	12.6
p2	0	51	120	1.67	43.03	110	60	1	1	8.3	4.64	1.08	5.9	15.8
p3	1	65	141.8	1.58	57	150	70	1	0	6.1	4.07	0.88	5.2	16
p4	1	42	153	1.74	51	130	80	1	0	4.5	4.19	1.11	5.6	14.2
p5	0	37	202	1.76	65	120	70	0	0	7.8	3.69	1	6.3	13.7
p6	0	55	136	1.79	43	150	90	1	1	4.5	4.1	1.15	7.7	16.3
p7	1	23	129	1.74	42.6	130	70	1	1	7.5	4.77	0.81	5.1	13.5
p8	0	62	150	1.78	47	130	80	1	0	5.4	4.6	1.05	5.7	16.3
p9	1	22	139.6	1.59	55	120	90	0	0	5	3.92	0.77	4.8	13.2
p10	0	29	208	1.89	58.2	110	60	1	0	6.6	4.04	1.03	5.2	15
p11	1	52	152	1.6	59.3	120	70	1	1	7.79	NA	1.6	6.7	16.3
p12	1	57	113	1.52	48.95	110	70	1	1	4.1	3.99	1.01	7.25	12
p13	1	40	126	1.67	44.1	110	70	1	0	6	4.27	0.86	5.2	14
p14	0	57	125.2	1.75	41	140	90	1	0	6.5	4.46	1.21	5.5	14.5
p15	1	35	117.9	1.6	46	140	80	0	0	6.3	4.18	1.19	5.5	13.6
p16	0	20	168	1.85	47	110	60	1	0	7.5	4.49	0.91	5.7	14
p17	0	67	136	1.64	68	110	90	1	1	7.7	3.77	0.98	8.8	14
p18	1	47	184.6	1.66	67.1	110	60	1	0	6.2	4.17	0.82	5.7	13.8
p19	0	57	200	1.68	70.9	130	70	1	1	6.1	4.08	1.35	6.2	14.2
p20	1	42	118	1.7	40.8	120	80	1	1	3.3	4.28	1.06	6.1	13.6
p21	1	64	120	1.56	49.3	140	80	1	1	0.28	4.4	1.03	NA	13.3
p22	1	58	103	1.54	43.43	120	80	1	1	4.4	4.9	1.09	6.4	12.9
p23	1	40	103	1.64	37.8	110	60	0	0	4.19	3.88	0.92	5.2	13.5
p24	1	61	132	1.64	48.8	120	80	1	0	4.8	3.75	1	5.8	14
p25	1	37	112	1.62	42.5	120	70	0	1	3.8	4.24	1.4	5.6	15
p26	1	23	148	1.5	65.7	140	80	1	0	5.6	4.25	0.9	5.6	14.3
p27	1	54	143	1.6	56	110	70	0	0	5.9	3.88	0.87	6.3	14.5
p28	1	64	113	1.52	48.6	120	80	0	1	5.5	3.91	0.97	6.4	14.1
p29	1	51	87	1.49	38.7	110	60	0	0	NA	NA	NA	NA	15.3
p30	1	39	212	1.6	84.9	120	60	0	1	5.3	3.73	0.65	5.6	12.7
p31	1	46	128	1.6	50	100	50	0	0	4.2	3.3	0.8	5.2	13.7
p32	1	60	96	1.52	41.55	120	80	0	1	NA	4.01	1.06	6.4	13.6
p33	1	52	119	1.55	49.5	120	80	1	1	6.3	4.41	0.92	6.5	13.5
p34	1	36	128	1.58	51.3	140	80	0	0	2.83	3.81	1.06	5.5	12.5
p35	0	41	153	1.73	51.1	130	80	1	0	6.97	4.48	1.25	6	12.4
p36	1	44	122	1.59	48.4	110	70	0	0	6.6	4.12	0.97	5.9	14.2
p37	1	56	150	1.52	65	110	50	0	1	6.8	4.46	1.48	6.1	13.3
p38	1	29	132	1.72	44.7	130	70	0	0	5.2	3.9	0.94	5.4	13.4
p39	1	46	99	1.56	40.7	120	60	0	0	5.3	4.7	0.93	5.7	12.4
p40	1	27	174	1.67	61	120	60	0	0	4.7	4.08	0.72	5.3	12.5
p41	1	46	197	NA	NA	130	70	1	1	5.4	4.05	0.96	5.5	14.2
p42	1	43	133	1.76	53	130	80	1	0	3.9	4.12	0.86	4.8	14.5
p43	1	43	144	1.57	58.5	110	60	0	1	6.4	4.34	NA	5.1	14.1
p44	1	48	143	1.6	55	110	70	1	1	5.1	4	0.86	5	13
p45	1	47	138	1.74	46	100	70	1	1	5.8	4.29	1.07	6.9	12.5
p46	1	40	101	1.64	37	NA	NA	0	0	3.8	4.24	1.34	4.9	11.9
p47	0	32	140	NA	41.8	120	80	1	0	6.4	4.52	1.28	6.4	15.1
p48	1	54	82	1.52	35.5	100	60	0	1	3.9	NA	1.03	5.1	12.5

*wg* weight (Kg), *hg* height (m), *BMI* body mass index, *BP sis* systolic blood pressure, *BP dias* diastolic blood pressure, *HT* previous diagnosis of hypertension, *DM2* previous diagnosis of diabetes mellitus 2, *Uri* uricemia, *Alb* albumina, *Glu* fasting glucose levels, *Hb1Ac* hemoglobin 1Ac, *HB* hemoglobin.

### Bariatric Surgery

Bariatric Surgery, also referred to as weight loss and metabolic surgery, is a medical intervention designed for individuals with obesity at elevated risk of morbidity and mortality. This procedure is reserved for those who have been unable to attain sufficient weight loss through lifestyle measures (such as diet, behavioral changes, and increased physical activity) and medical treatments and who are experiencing complications associated with obesity.

Sleeve gastrectomy and gastric bypass are currently the two most frequently performed procedures. The former involves removing a large portion of the stomach, leaving a smaller "sleeve" or tube-shaped stomach. The reduced stomach size limits the amount of food that can be consumed, and it also affects hunger-regulating hormones. In the latter, a small pouch is created at the top of the stomach and connected directly to the small intestine. This reduces the amount of food the stomach can hold and bypasses part of the small intestine, reducing the absorption of calories and nutrients. Both of these procedures are routinely done in the present program.

### FSTL1 ELISA measurements

Blood collected from patients was centrifuged at 1000×*g* for 10 min at room temperature. The plasma was carefully removed and dispensed into 1 ml aliquots, then stored at − 80 °C until further analysis. FSTL1 concentrations were measured in undiluted plasma using the Human FSTL1 ELISA Kit (ab213782). Two measures were taken per patient: at program entry and after six months of surgery. Venous blood samples were taken in fasting.

### Linear models and statistical analysis

We used R statistical software (R-project) for statistical analysis (www.r-project.org).

The linear models were constructed iteratively, adding one independent variable at each iteration, among the following relevant ones: age, weight, height, BMI, difference in systolic and diastolic arterial pressure (PAdif), hemoglobin, platelets, crea, fasting glucose level, HBA1C, Uricemia, total cholesterol, LDL, HDL, Triglycerides, ALT, GPT, AST, GOT, Albumin, TSH, and FSTL1 values pre-surgery.

The dependent variable (BS success) used in every model was the difference in BMI before and after the surgery. For each model, we calculated the BIC (Bayesian information criterion, which is a criterion for model selection among a finite set of models; models with lower BIC are generally preferred) and evaluated the p-value, R-squared, adjusted R-squared, and the model coefficients with their corresponding p-value.

We analyzed all linear models with one to 10 independent variables and selected the best model according to the BIC. We kept the model with (all) statistically significant coefficients, a significant model p-value, and the higher adjusted R-squared.

To evaluate overfitting (usually models with many parameters adjusted from a small data set are prone to overfitting), we used a leave-one-cross-validation.

To analyze the physiological relevance of FSTL1, we determined the Spearman correlation of FSLT1 levels in two time points (pre-surgery and six months after) with different clinical variables before and after surgery.

Differences in means for continuous variables were calculated with t-tests.

The tendency test used here was the Mann–Kendall tendency test. It was used to evaluate FLST1 tendencies pre and post surgery.

## Results

### Baseline characteristics of the cohort

We assessed 48 patients involved in the obesity program of the Maciel University Hospital in Uruguay. We measured several clinical features at program entry (Table 1, Supplementary Table S1, Supplementary Figs. S1 and S2).

Most patients were women (37, 77%), with a median age of 46 years and a mean BMI of 51 (Fig. S1). Of the 48 individuals, 21 (43.8%) had a previous diagnosis of diabetes mellitus (DM). Also, 27 (56.3%) had a prior diagnosis of hypertension (HT) and were taking blood pressure medication. Notably, the median fasting glucose value was 1, likely because 52% of the individuals took metformin then.

Six patients also had previous cardiac pathology. The median Hemoglobin 1Ac (HBA1c) value was 5.7 (Fig. S1), and the median values of LDL, HDL, and TG were 94.5, 45, and 126, respectively (Fig. S1). The median platelet value was 249 500, within the normal range (150,000–400,000).

### Weight loss and comorbidities after follow-up

**Figure 1:**
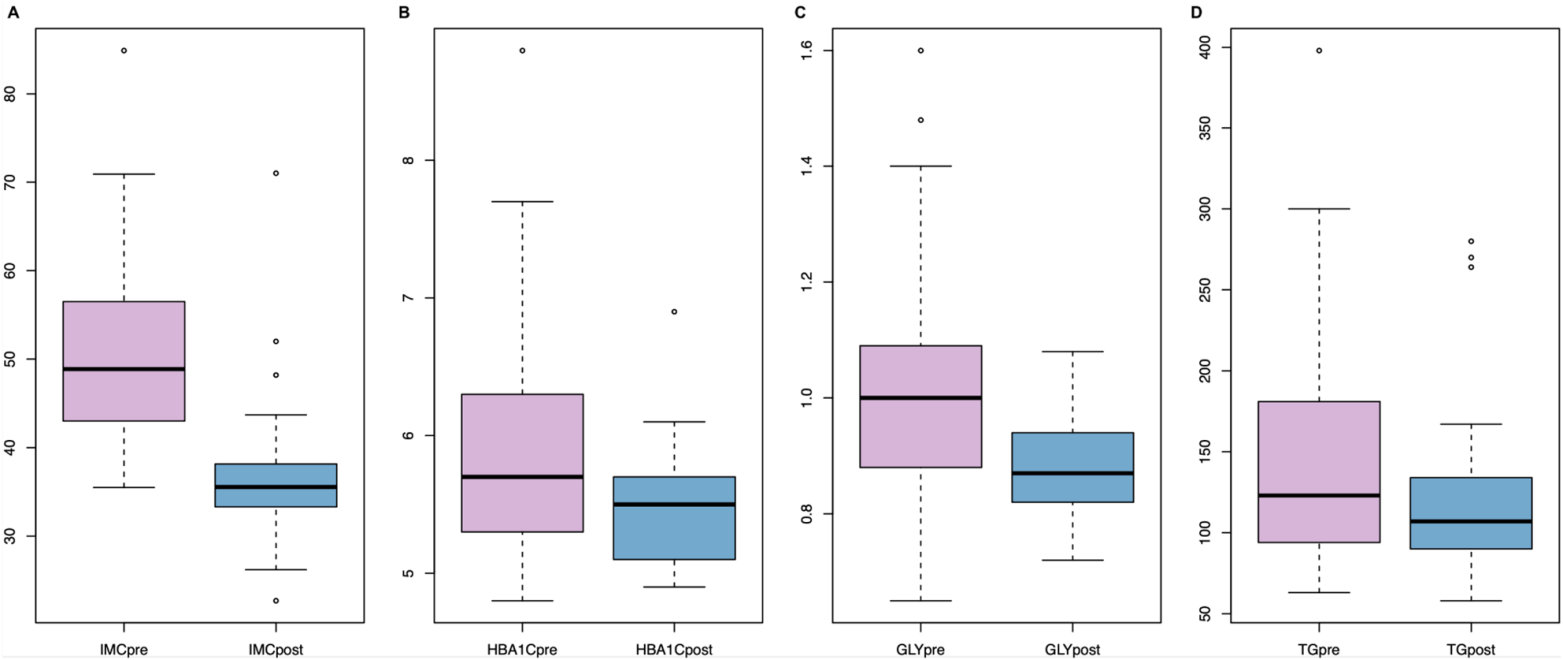
Continuous variables previous and after surgery. (**A**) Body mass index (**B**) Hemoglobin 1AC, (**C**) Glycemia, (**D**) Triglycerides.

Six months after Bariatric Surgery, clinical variables were reassessed. Notably, almost all clinical features relevant to cardiometabolic health were significantly improved after BS. Of the patients who came for follow-up (28), we compared their BMI with their initial pre-surgery BMI. The mean BMI of the pre-surgery group was 51, and the post-surgery group was 36.27 (p-value = 2.342e−09, Fig. 1A). We also compared HBA1C (Fig. 1B), Glycemia (Fig. 1C), and TG (Fig. 1D) pre-and post-surgery, among other variables (Fig. S3). HBA1C, TG, and Glycemia post-surgery reductions were significant (p-value = 0.001963, 0.03929, and 0.002, respectively). The increase of HDL and the decrease of ALT.GPT was only marginally significant (Fig. S3, p-value = 0.06 and 0.062, respectively).

We also evaluated HT after surgery by analyzing the consumption of hypertensive prescription drugs. Only 5 of the 28 kept the consumption of hypertensive medications. Only one individual was classified as diabetic after surgery.

### Change in FSTL1 levels and correlation with clinical variables

We measured FSTL1 plasma levels before (pre) and six months (6 M) after the surgery for 28 patients.

**Figure 2:**
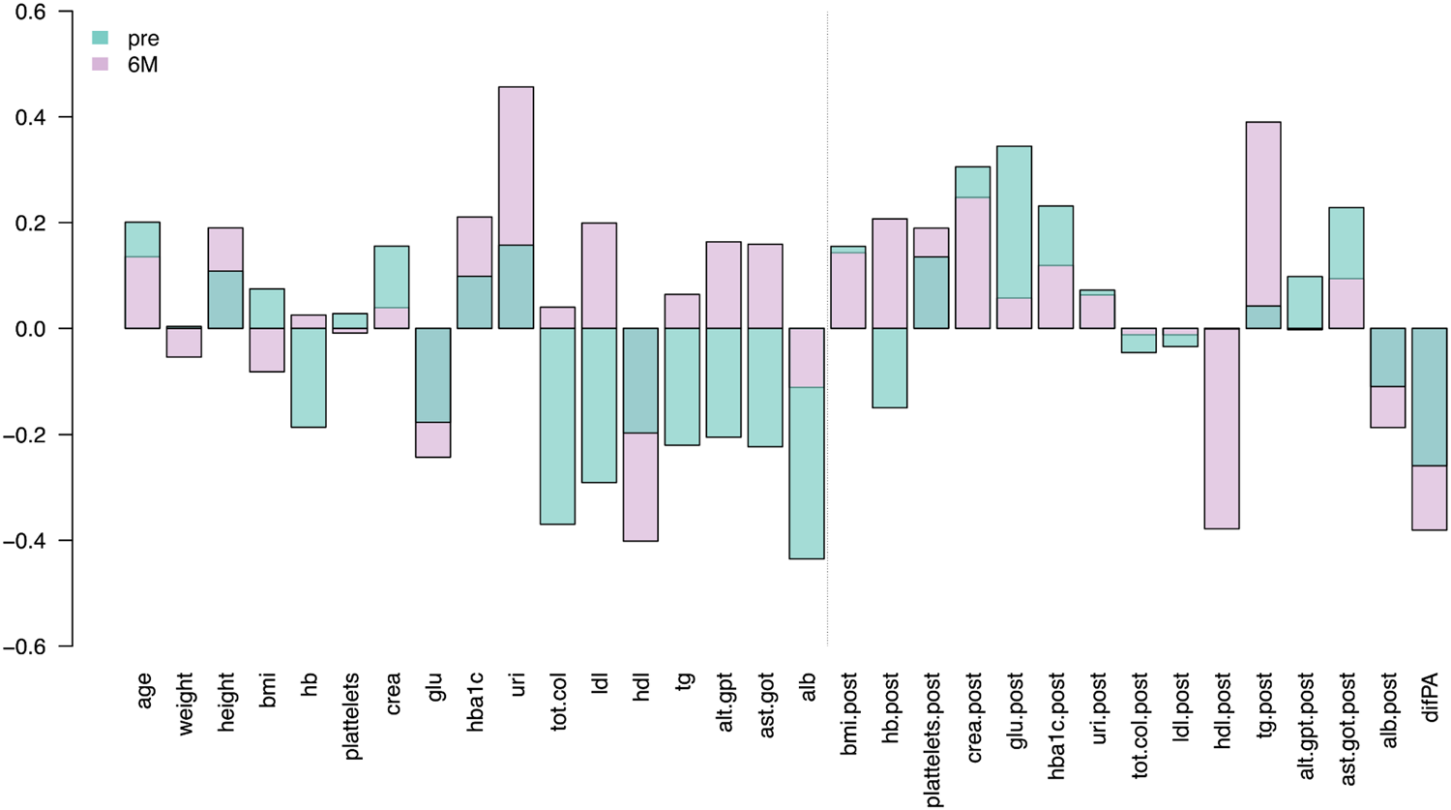
Correlation of clinical variables with FSTL1 values at different time points: pre-surgery (Pre) and six months after the procedure (6 M). *hb* hemoglobin, *Glu* fasting glucose, *uri* uricemia, *col.tot* total cholesterol, *tg* triglycerides, *ALT.GPT* AST.GOT, *alb* albumin.

FSTL1 values pre-surgery significantly correlate with total cholesterol and Albumina pre-surgery values, with − 0.37 (p-value 0.052) and − 0.44 (p-value 0.02) (Fig. 2). Also, FSTL1 pre-surgery values correlated (only marginally significant) with the fasting glucose levels after surgery: 0.34 (p-value 0.09), which means that for high values of FSTL1, we observed high fasting glucose levels after surgery. Additionally, FSTL1 values six months after surgery correlate with Uricemia and HDL values pre-surgery, with values of 0.46 (p-value 0.03) and − 0.40 (p-value 0.052), respectively (Fig. 2). It also correlated (only marginally significant) with post-surgery values of HDL with − 0.3780 (p-value 0.09) and Triglycerides with 0.390 (p-value 0.08).

To sum up, high FSTL1 levels tend to be more associated with metabolic syndrome and an inflammatory state, but it does not necessarily correlate to BMI or BS success alone. Hence, we analyzed linear models to include FSTL1 and several clinical variables to explain BS success.

### Linear models suggest an influence of FSLT1 in Bariatric Surgery success as measured by BMI difference

**Figure 3:**
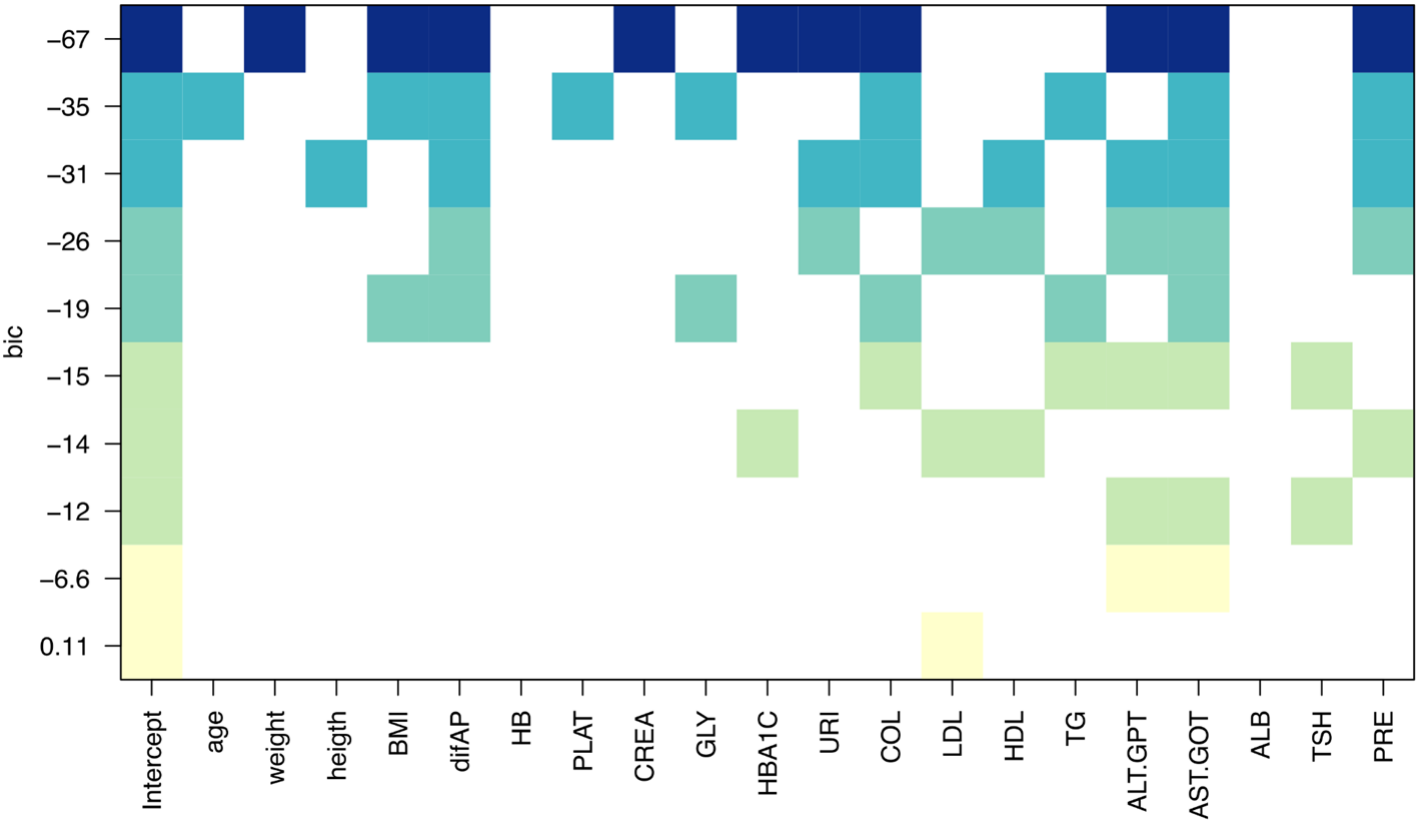
Linear models. Best models of one to ten variables as measured by BIC. The X-axis shows independent variables iteratively entering the model (colored squares mean they are within the model). Y-axis, BIC of each model.

The best model had ten variables with a BIC of -63 (Fig. 3). Coefficients and p-values are shown in Table 2. The adjusted r^2^ value and p-value for the complete model were 0.95 and 6.37e^−6^, respectively. Adjusted r^2^ values and p-values for the other models are shown in Fig. S4A and B, respectively. Using the model, we predicted the values of difference in BMI and analyzed the differences between the actual data and the predicted one using the model; the differences ranged from − 1.0 to 0.58 in BMI. Thus, the proposed model could predict the BMI differences accurately, with a deviation of maximal 1; as a reference, in a person with height 1.63 (mean height of the present cohort), this would imply a maximal error in the prediction of 2.65 kg.

**Table 2:** Coefficients and p-values of the best linear model according to BIC.

	Estimate	P-value
Intercept	− 35.60	1.84^e−7^
Weight	0.15	1.28^e−6^
Difference AP	0.25	1.35^e−7^
BMI before	− 0.45	1.90^e−7^
CREA	− 14.01	1.01^e−5^
HBA1C	3.78	7.74^e−7^
URICEMIA	1.68	2.85^e−6^
CHOLESTEROL	− 0.06	1.01^e−6^
ALT.GPT	− 0.31	2.22^e−7^
FSTL1 before	− 0.27	6.20^e−8^
AST.GOT	0.49	7.62^e−8^

A leave-one-cross-validation yielded an R-squared of 0.41, an RMSE of 4.35, and an MAE of 3.13. Therefore, rather than focusing on the exact model (due to small dataset limitations), we sought to determine the variables that are influencing the outcome stably, e.g., the variables that, once they enter the model, remain within the model when adding an additional variable (Fig. 3). To do this, we reformulated the model with fewer ‘stable’ variables, including difAP, COLESTEROL, and ALT.GPT, AST.GOT and PRE (pre-surgical FSTL1 level). Coefficients and p-values are in Table S2. Adjusted R^2^ is 0.65, and the model's p-value is 0.00040. We formulated a significant linear regression model that captures relevant clinical and biological variables influencing the surgery’s success.

Additionally, we observed a tendency for FSTL1 levels to decrease after the surgery event.. Results indicated a negative trend in the difference between pre-surgical levels of FSTL1 and six months after the surgery (tau = − 0.423, with a p-value of 0.005118).

These results suggest that clinical variables related to blood pressure, cholesterol, hepatic enzymes, and FSTL1 levels correlate with the BS outcome. The higher the FSTL1 level (also CREA, AST, cholesterol, and BMI), the better the BS outcome in terms of BMI difference. Also, after surgery, FSTL1 levels have a significant tendency to drop.

## Discussion

This study of Uruguayan BS patients confirmed that BS significantly reduces BMI and alleviates most obesity-related metabolic complications as previously reported. Also, our analysis revealed that pre-surgery FSTL1 levels significantly correlate with total cholesterol and albumin pre-surgery, and marginally with fasting glucose levels post-surgery. Six months post-surgery, FSTL1 levels correlate significantly with pre-surgery uricemia and HDL values, and marginally with post-surgery HDL and triglycerides, suggesting an association with metabolic syndrome and an inflammatory state rather than BMI or BS success alone. Moreover, higher FSTL1 levels, along with CREA, AST, cholesterol, and BMI, are associated with better BS outcomes in terms of BMI difference, and FSTL1 levels significantly tend to drop after surgery.

Of the 21 patients with diabetes at program entry, we were able to assess HBA1C at six months after surgery in 15 patients. Of those, 14 had HBA1C values lower than 6.5, implying a diabetes remission of 93%^20^. Nonetheless, the ‘unremitted’ patient had insulin dependency before the procedure. The other patient with insulin dependence no longer needed insulin after surgery and remitted fully. Fasting glucose levels were below 1.08; the mean value was 0.89 without anti-diabetic drugs (but the insulin-dependent patient). Previous reports in other populations highlighted improvements in type 2 diabetes, revealing consistent results in the percentage of patients achieving long-term remission from the disease^21–23^.

Cholesterol levels also improved after the surgery. Specifically, triglyceride levels decreased significantly after surgery. The range of blood triglycerides after weight-loss surgery was from 58 to a maximum of 280, with a median of 109, and only one patient was still on cholesterol drugs. Also, previous studies show improvements in cholesterol levels after surgery that are compatible with our findings^20,21^.

In general, as seen in other reports, BS improves several cardiometabolic variables significantly in the short, medium (as shown here), and long term. Additionally, liver enzymes such as Alanine Aminotransferase (ALT) and Aspartate Aminotransferase (AST) are also measured in such studies. That study shows a significant decrease of both enzymes after surgery^24^, which goes in the same line as our findings (Fig. S3); we also reported a − 0.31 coefficient of ALT enzyme in the linear model describing the success of the surgery (3.3), meaning that the increase of one unit of ALT implies an increase of 0.36 in the differences of BMIs. The potential impact could be more pronounced post-surgery in individuals with a less healthy condition before the procedure. Surprisingly, AST enzymes had a positive coefficient, meaning that an increase in AST decreases the difference in BMIs.

The same holds for total cholesterol, however, with a very low impact on the dependent variable (coefficient of − 0.06). Creatinine had a high impact (coefficient − 14.01) on the outcome, implying that augmentation of one unit of Creatinine has a large increase of 14 in the BMI difference. However, the range of Creatinine is 0.45 to 1.44, indicating that an increase of one unit likely has a large impact at the clinical level.

In addition to classical clinical parameters, we also investigated FSTL1 levels in our cohort and their potential involvement in the success of Bariatric Surgery. Other studies have already proposed that FSTL1 plays a role in obesity, although its role in the pathogenesis of the disorder or its presentation has not been fully elucidated yet.

Here, we observed a negative significant correlation between the FSTL1 values and albumin pre-surgery, meaning that higher values of FSTL1 would suggest lower values of albumin. Decreased albumin levels have been linked to higher body fat percentage and inflammation markers in adipose tissue, such as macrophages. This association suggests that reduced albumin concentrations may be connected to the inflammatory processes associated with the development of obesity^25^. In this context, higher levels of FSLT1 might be correlated with obesity and inflammation. We did not observe a correlation between FSTL1 levels and BMI in our work.

As mentioned, higher levels of FSTL1 have been linked to a more unhealthy presentation of obesity^19^. Interestingly, we did observe a tendency for FSTL1 to decrease after the surgery event, thus potentially contributing to the success of the surgical intervention in regulating the different metabolic parameters. Importantly, our data also shows that higher FSTL1 levels contribute to a better outcome of the surgery (increased BMI reduction). In our linear models, a one-unit increase in FSTL1 (PRE-surgery) accounts for a 0.27 increase in the difference of BMIs, again measured as BMI post–BMI pre (consistently negative). Thus, our results could imply that more severely affected obese patients likely obtain a more significant benefit from BS. Importantly, FSTL1 levels had a more significant impact on our prediction model than BMI. Hence, our results would argue that the patients who benefit the most from surgery are those with a more pronounced metabolic dysfunction and not necessarily those who are more obese. In summary, with this study, and despite being limited by the number of patients, we have identified factors influencing BS success that need further investigation with larger data sets. In addition, our results might indicate that including FSTL1 levels in the pre-surgery evaluation could be helpful in decision-making and patient management in the clinic.

We performed two types of surgeries in this study (gastrectomy sleeve and gastric bypass), and due to the small sample size, we were not able to discriminate the analysis by surgery type, which might be interesting to understand whether there are different success rates. Also, we acknowledge that adherence to the study was not ideal (28 from 48 patients) for long-term follow-up (6 months after surgery).

## Conclusion

This study of Uruguayan BS patients confirmed that BS significantly reduces BMI and alleviates most obesity-related metabolic complications. Our analysis revealed significant correlations between pre-surgery FSTL1 levels and total cholesterol, albumin, uricemia, and HDL values, indicating a link between FSTL1 and metabolic syndrome and inflammatory states. Higher FSTL1 levels, CREA, AST, cholesterol, and BMI were associated with better BS outcomes, and FSTL1 levels significantly dropped post-surgery. Additionally, we observed a high rate of diabetes remission and significant improvements in cholesterol and triglyceride levels, aligning with previous studies.

Our results suggest that incorporating FSTL1 levels into pre-surgery evaluations could enhance decision-making and patient management, potentially offering a more personalized approach to Bariatric Surgery. Despite the study's limitations, including a small sample size, varied surgery types and varying adherence rates, these insights contribute to understanding the complex factors influencing BS success.

### Supplementary Information

Supplementary Figures.

Supplementary Table S1.

Supplementary Table S2.

## Data Availability

The complete table with the clinical metadata and biomolecular measurements are available upon reasonable request (contact corresponding author). The rest of data generated and analyzed during this study are included in this published article [and its supplementary information files].
