# Supplementary material for: Impact of Bariatric Surgery on metabolic health in a Uruguayan cohort and the emerging predictive role of FSTL1
Source: Sci Rep. 2024 Jul 2;14:15085. doi: 10.1038/s41598-024-65651-8 (PMC11219826; doi:10.1038/s41598-024-65651-8)
Supplement: Supplementary file 1 — Supplementary Figures. [file 41598_2024_65651_MOESM1_ESM.pdf]

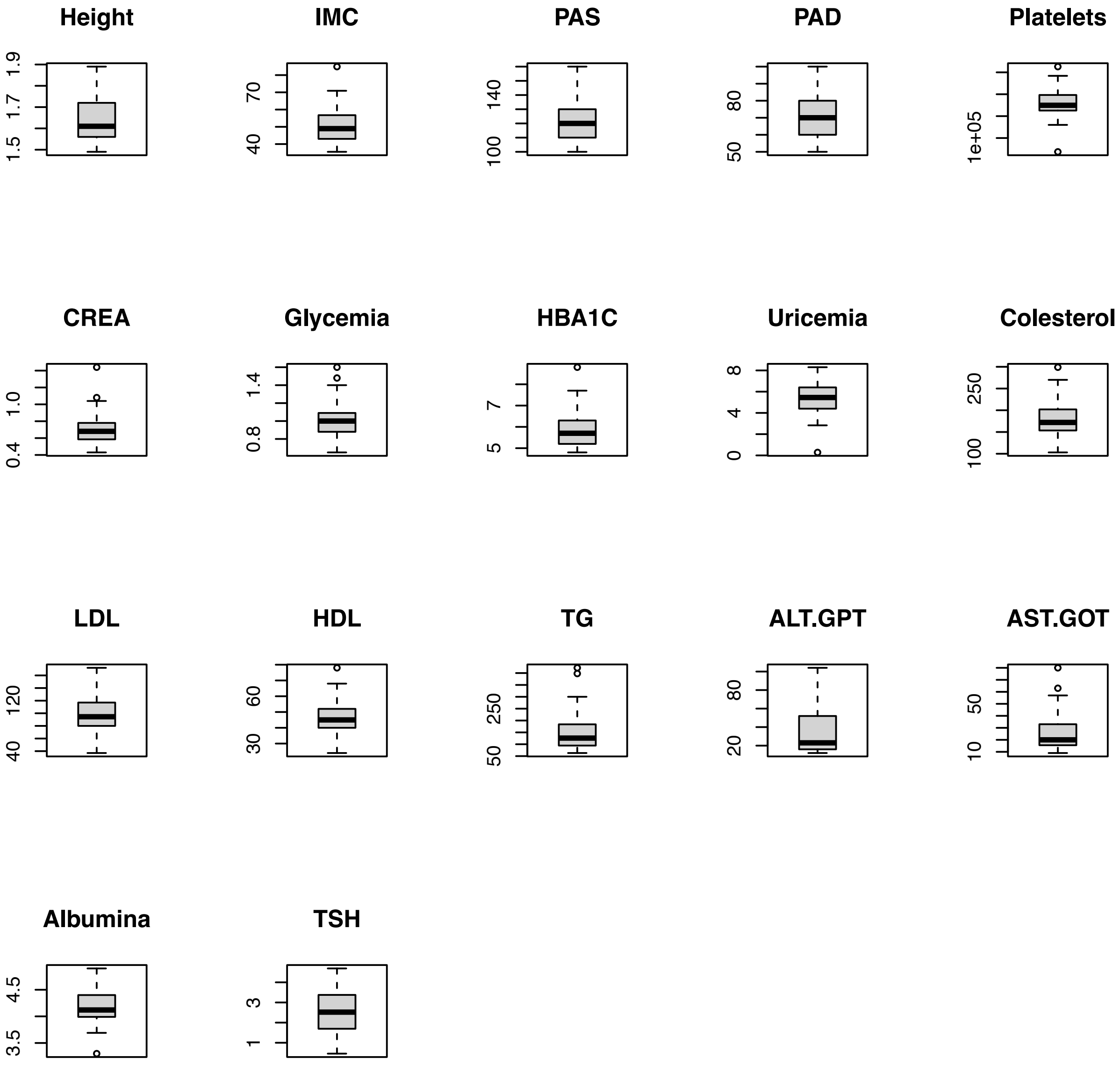

Figure S1: Distribution of continuous clinical variables at program entry

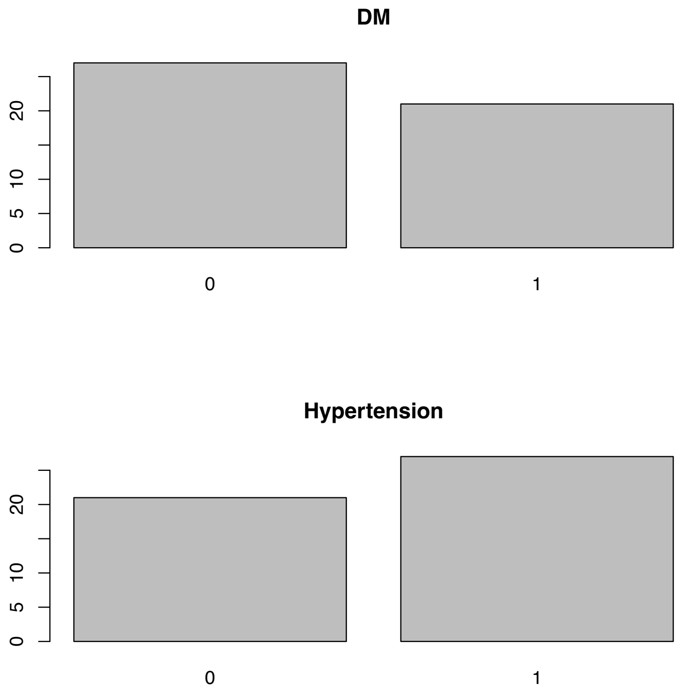

Figure S2: Distribution of categorical variables at program entry. **A:** Diabetes mellitus. **B.** Hypertension.

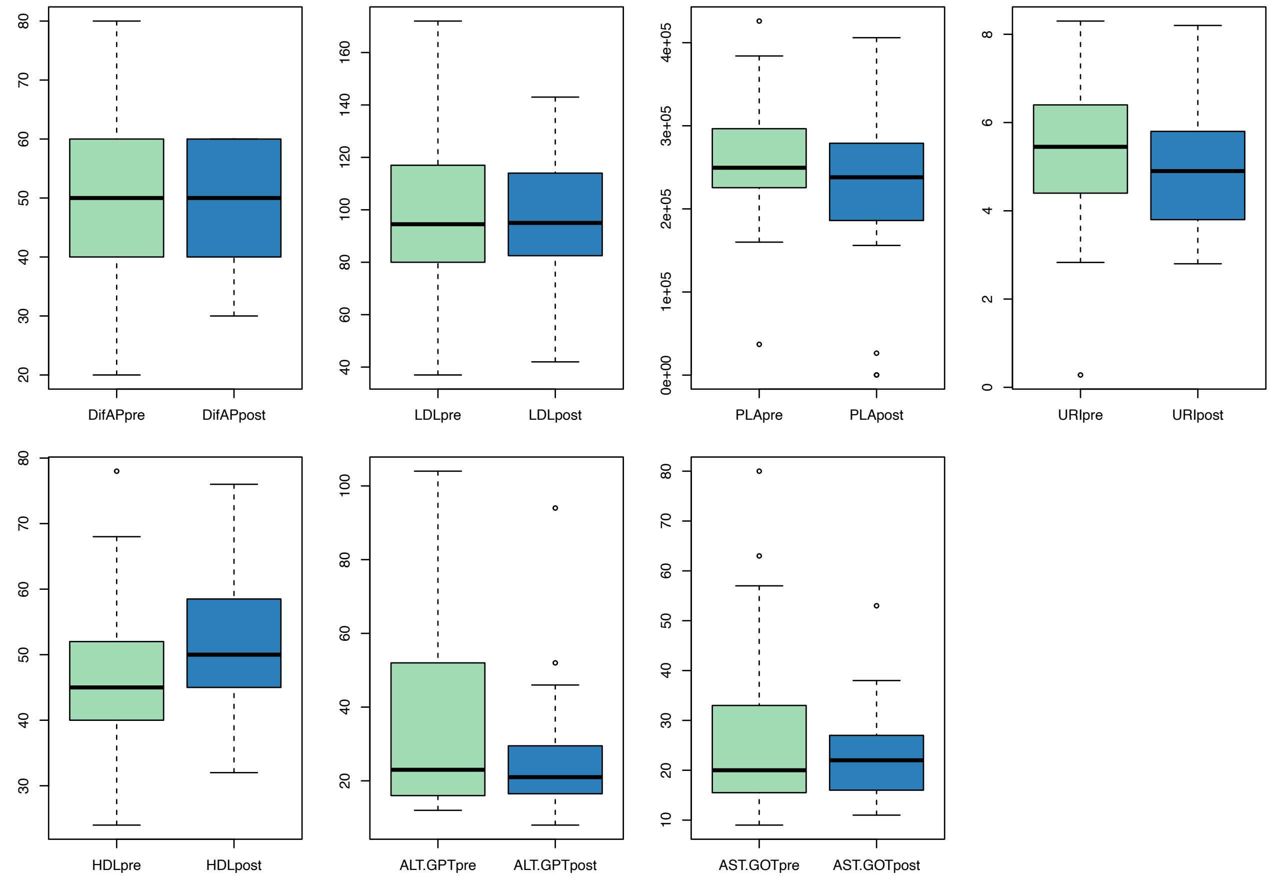

Figure S3: Distribution of continuous variables pre and post-surgery.

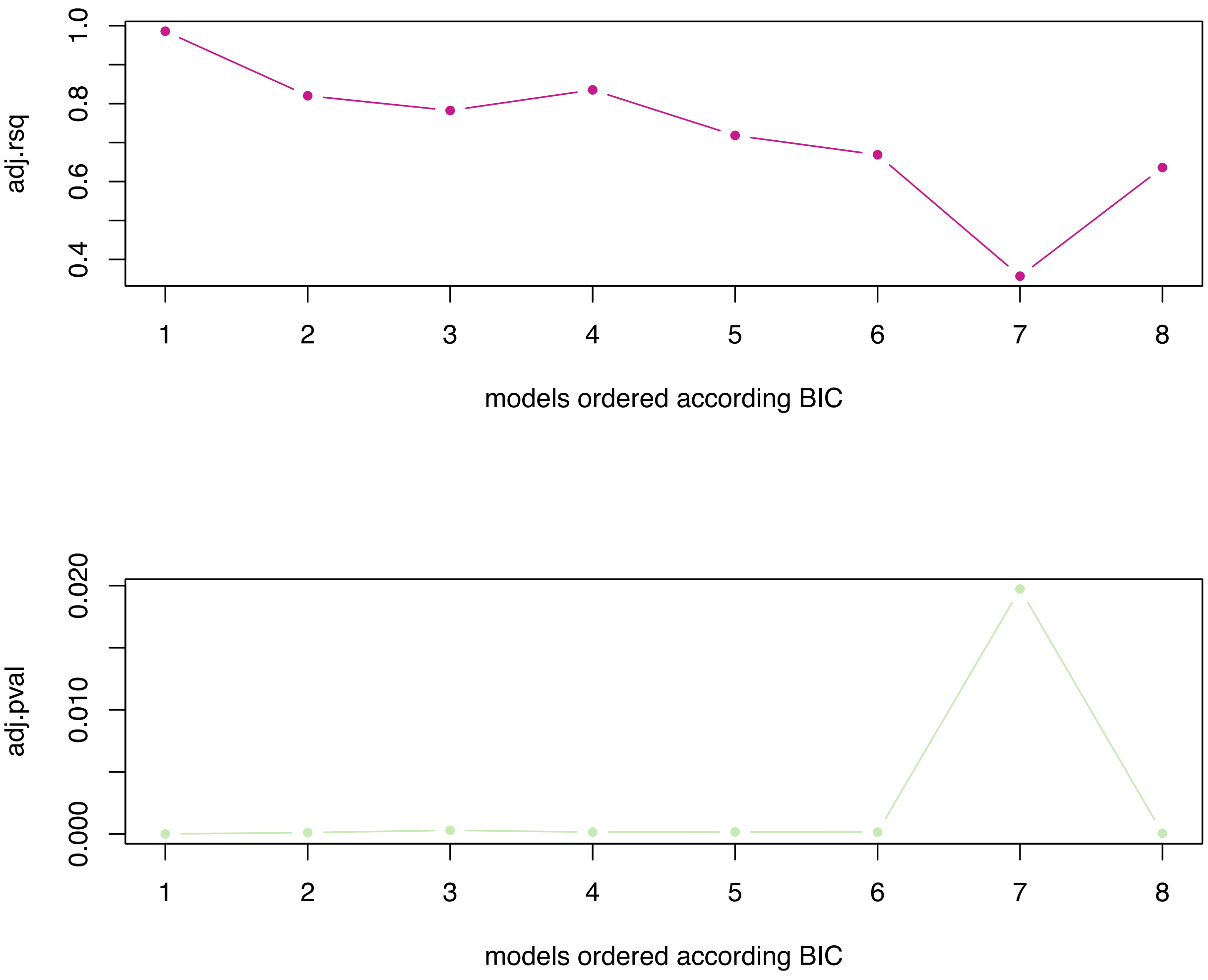

Figure S4: Adjusted R-squared and p-values of all models.
